# Supplementary material for: Room-temperature waveguide-integrated photodetector using bolometric effect for mid-infrared spectroscopy applications
Source: Light Sci Appl. 2025 Mar 19;14:125. doi: 10.1038/s41377-025-01803-3 (PMC11920593; doi:10.1038/s41377-025-01803-3)
Supplement: Supplementary file 1 — Supplementary Information [file 41377_2025_1803_MOESM1_ESM.docx]

Supplementary Information for

Room-temperature waveguide-integrated photodetector using bolometric effect for mid-infrared spectroscopy applications

Joonsup Shim^1^, Jinha Lim^1^, Inki Kim^1^, Jaeyong Jeong^1^, Bong Ho Kim^1^, Seong Kwang Kim^1^, Dae-Myeong Geum^2^, and SangHyeon Kim^1*^

^1^School of Electrical Engineering, Korea Advanced Institute of Science and Technology (KAIST), 291 Daehak-Ro, Yuseong-Gu, Daejeon 34141, Republic of Korea

^2^Department of Electrical & Computer Engineering, Inha University, 100, Inha-ro, Michuhol-gu, Incheon 22212, Republic of Korea

*^*^Corresponding Author: SangHyeon Kim (shkim.ee@kaist.ac.kr)*

**Note 1.** Free-carrier absorption (FCA) and two-photon absorption (TPA) in Ge

**Note 2.** Thermal conductivity analysis of yttrium oxide (Y_2_O_3_) layer

**Note 3.** Optimizing device geometries

**Note 4.** Fabrication process flow

**Note 5.** Device characterization

**Note 6.** High-temperature stability

**Note 7.** Low-frequency noise analysis

**Note 8.** Electrical breakdown characteristics

**Note 9.** Propagation loss of waveguides

**Note 10.** Simulation of mode converters

**Note 11.** Performance comparison

**References**

**Supplementary Note 1. Free-carrier absorption (FCA) and two-photon absorption (TPA) in Ge**


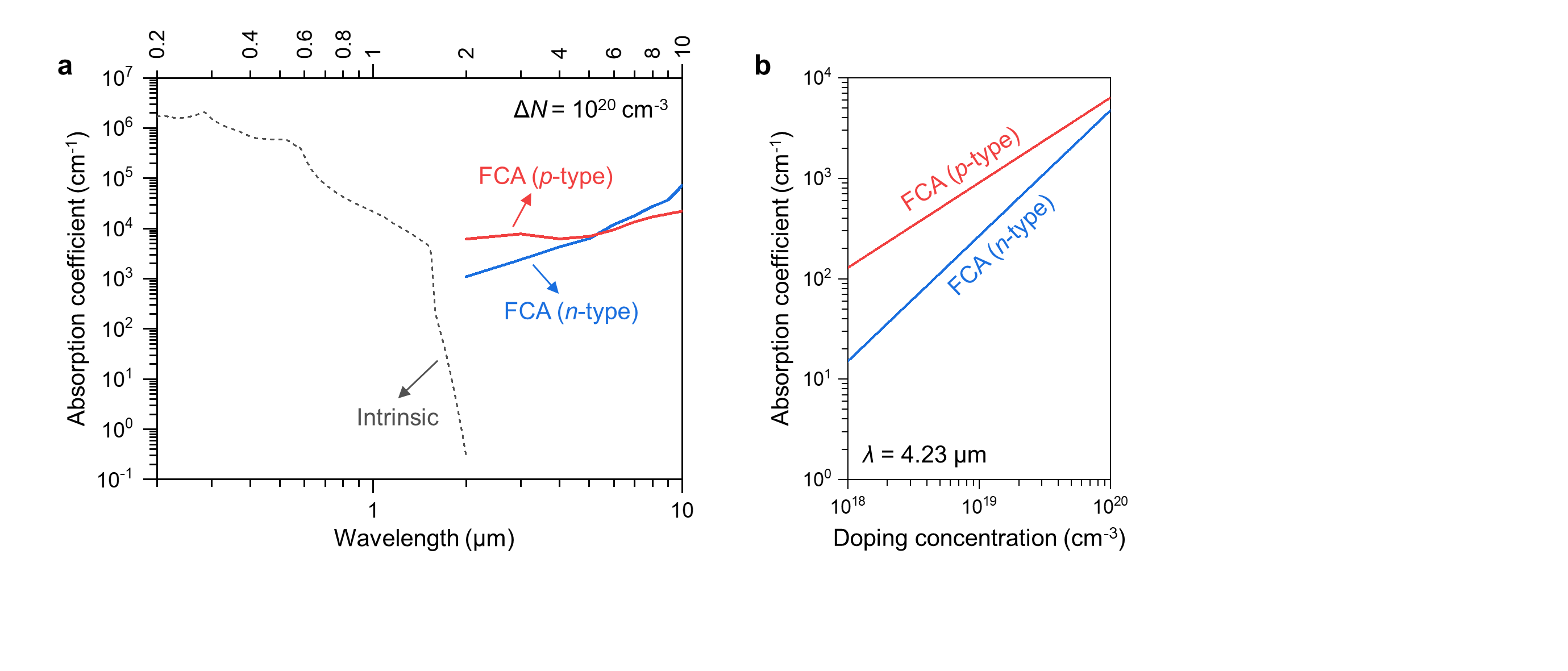


Fig. S1. FCA in Ge. a Absorption coefficients in Ge for intrinsic absorption and for FCA in both *n*- and *p*-type Ge with a doping (impurity) concentration (Δ*N*) of 10^20^ cm^-3^ depending on the wavelengths. b Comparison of the calculated FCA coefficients as a function of the doping concentration for *n*- and *p*-type Ge at a wavelength (*λ*) of 4.23 μm. The absorption coefficients for FCA in Ge are calculated based on the literature^1^.

The free-carrier absorption (FCA) is a process where free carriers, i.e., free electrons and free holes, absorb the incoming photon energy, leading to non-radiative carrier transition with the thermalization process^1,2^. There are two types of FCA in Ge: FCA in *n*-type Ge and FCA in *p*-type Ge, each corresponding to the absorption attributed to the free electrons and free holes, respectively. The absorption coefficients in Ge for intrinsic absorption (band-to-band absorption) and FCA are plotted in Fig. S1a. The doping (impurity) concentration (Δ*N*) is assumed to be 10^20^ cm^-3^. The intrinsic absorption of Ge is negligible beyond the wavelength of ~1.9 μm; however, FCA becomes increasingly significant beyond the bandgap energies. Furthermore, FCA in Ge shows a clear trend of increase with the wavelength of light, highlighting the importance of our approach for broadband mid-infrared (MIR) photodetection – even much higher photoresponse at longer wavelengths – within the waveguide structures on the Ge-on-insulator (Ge-OI) platform. Figure S1b represents the absorption coefficients of FCA for *n*- and *p*-type Ge depending on the doping concentration at the wavelength of 4.23 μm, chosen for the carbon dioxide (CO_2_) gas sensing demonstration in this work. As shown in Fig. S1b, FCA is more pronounced in *p*-type doped Ge compared to *n*-type. Consequently, we selected *p*-type doping to maximize the FCA-induced heating process in our detector.

The two-photon absorption (TPA) is a nonlinear optical process where an electron simultaneously absorbs two photons to transition from a lower energy state to a higher energy state. When the photon energy is less than half the bandgap energy, the probability of TPA is significantly reduced. Ge has an indirect bandgap of ~0.66 eV. Since the photon energy in this work (beyond the wavelength of 4 μm) is still below the half of the bandgap energy, the TPA in Ge is significantly reduced or absent. However, TPA can play a role within the doped Ge region since doping introduces impurity energy levels within the bandgap, enabling TPA by providing intermediate states even at photon energies below half the bandgap energy. This can also generate free carriers at a rate proportional to the square of the optical intensity, leading to non-radiative recombination and releasing energy as heat. Here, since the optical power coupled into the bolometer region is much lower than the threshold intensity^3^, free-carrier absorption (FCA)-induced thermalization process is the dominant absorption mechanism. However, at intensities approaching or exceeding this threshold, TPA-induced heat generation can also contribute to the bolometric photoresponse in the proposed detector on the Ge-OI platform.

**Supplementary Note 2. Thermal conductivity analysis of yttrium oxide (Y_2_O_3_) layer**


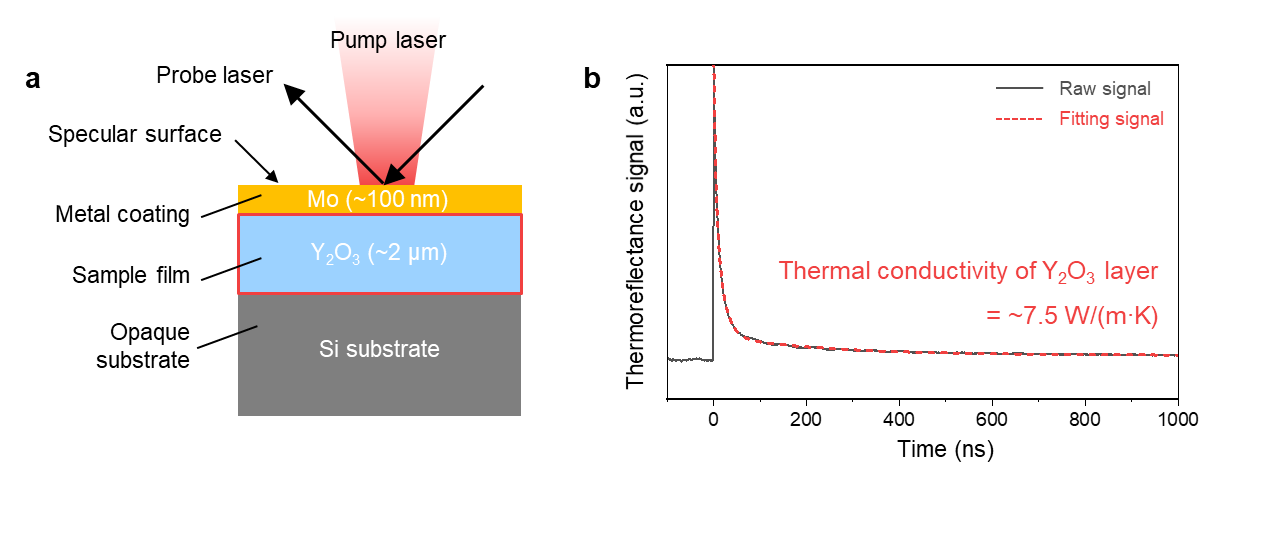


Fig. S2. Thermal conductivity analysis of an Y_2_O_3_ layer using a time-domain thermo-reflectance (TDTR) front heating/front detection (FF) technique. a Schematic diagram illustrating the TDTR FF method. b Normalized TDTR signal (black line) and the corresponding fitting curve (red dotted line) obtained using the mirror image method. The thermal conductivity of the Y_2_O_3_ layer was determined to be ~7.5 W/(m∙K).

To investigate the thermal conductivity of an yttrium oxide (Y_2_O_3_) layer prepared by the radio-frequency magnetron sputtering method in this work, we conducted a time-domain thermoreflectance (TDTR) analysis^4,5^ in the front heating/front detection (FF) configuration using a nano-second thermoreflectance apparatus (NanoTR, NETZSCH). This equipment system is a suitable solution for analyzing the thermal properties of thin layers and films, which typically differ from those of bulk materials. The FF measurement configuration can be applied to extract the thermal properties of thin layers on opaque substrates, whereas the rear heating/front detection (RF) method is more appropriate for the sample with transparent substrates.

Figure S2a illustrates a schematic diagram of the sample setup with the TDTR FF technique. The sample consists of a multilayer structure with a 100-nm-thick molybdenum (Mo) layer on top, serving as a transducer, followed by a 2-μm-thick Y_2_O_3_ layer, and a silicon (Si) substrate. The pump laser induces a transient thermal response, which is detected from the front side of the sample. Figure S2b shows the normalized TDTR signal obtained using the FF configuration. The temperature history curve in Fig. S2b reveals an instantaneous temperature rise due to the pump laser irradiation. Subsequently, the temperature gradually decreases as heat diffuses through the Mo layer, eventually reaching the boundary and beginning to diffuse into the Y_2_O_3_ layer. Using the mirror image method^6^ (fitting curve), we analyzed the behavior of decaying signal. The thermal conductivity of the Y_2_O_3_ layer was estimated to be ~7.5 W/(m∙K).

**Supplementary Note 3. Optimizing device geometries**


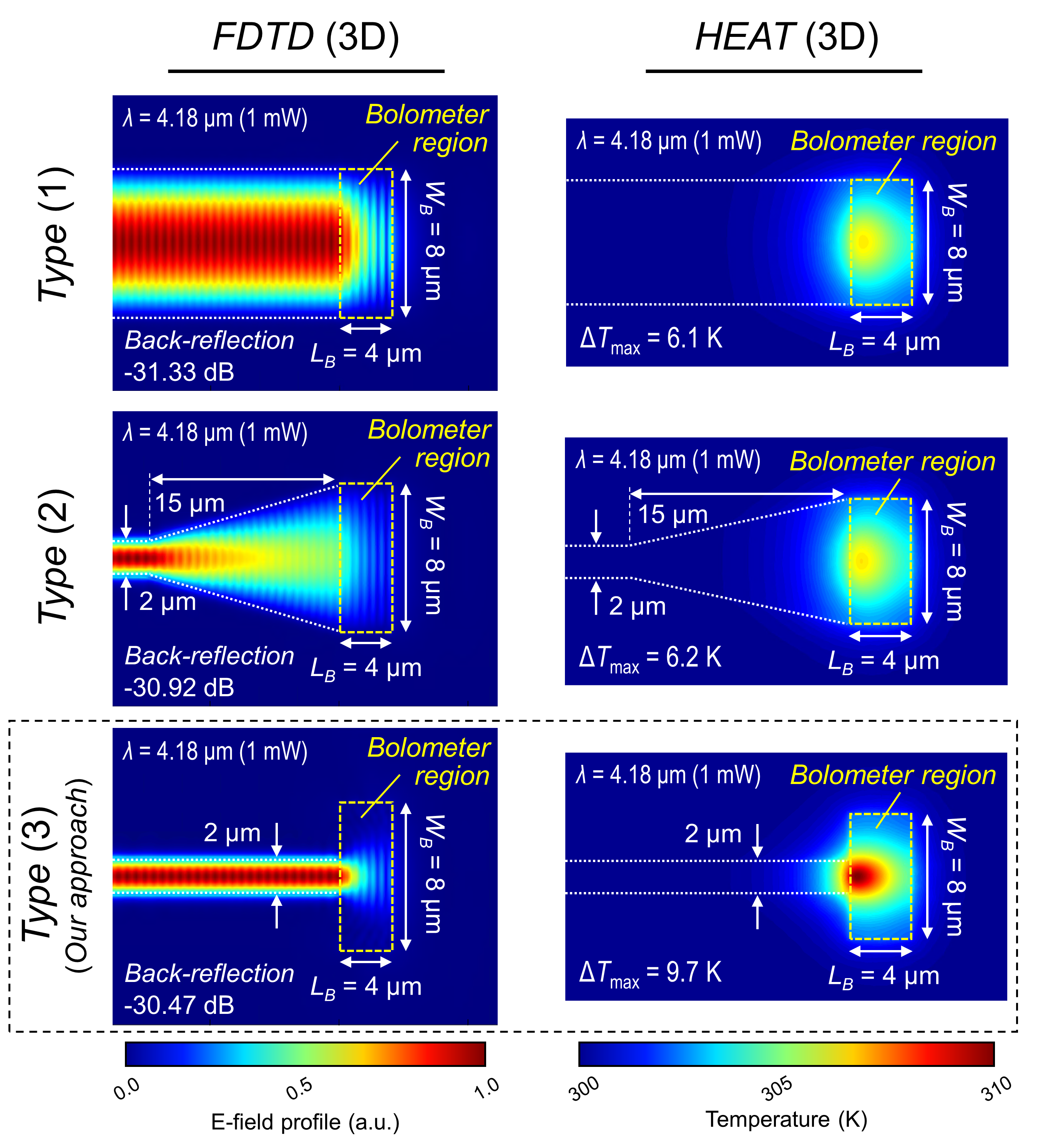


Fig. S3. Optimization of waveguide-to-bolometer interfacing geometries. The amount of back-reflection and FCA-induced heating efficiency was obtained through numerical simulations based on different interfacing geometries. Three device configurations were evaluated: Type (1) features a conventional structure, Type (2) incorporates a tapered structure, and Type (3) – our approach – utilizes an abrupt structure.

The optimization process of device geometries was conducted by numerical simulations of 3D-FDTD and HEAT solvers (Ansys Lumerical). We first introduced a terminated-waveguide structure, in which the input waveguide is terminated after the bolometer region (*p*^+^ Ge region). Furthermore, we carefully considered the waveguide-to-bolometer interfacing geometry. Figure S3 describes a comparative analysis of three device configurations depending on the interfacing geometries: type (1) – conventional structure, type (2) – tapered structure, and type (3) – abrupt structure (our approach). An input light of 1 mW at 4.18 μm was assumed to support only the fundamental transverse-electric (TE) mode. Here, we primarily considered the amount of back-reflection and the FCA-induced heating efficiency. Type (1) illustrates a conventional waveguide with a uniform width leading directly into the bolometer region, which shows a back-reflection of -31.33 dB and a maximum temperature increase (Δ*T*_max_) of 6.1 K. Type (2) depicts a tapered waveguide structure that gradually widens towards the bolometer region, resulting in a back-reflection of -30.92 dB and a Δ*T*_max_ of 6.2 K. Type (3) represents an abrupt transition design with a narrow waveguide expanding suddenly to the relatively wider bolometer region, producing a similar lower back-reflection of -30.47 dB and a notably higher Δ*T*_max_ of 9.7 K compared with other approaches. Consequently, we opted for the type (3) – abrupt structure – as our approach for enhanced FCA-induced heating performance with reasonable amount of back-reflection.


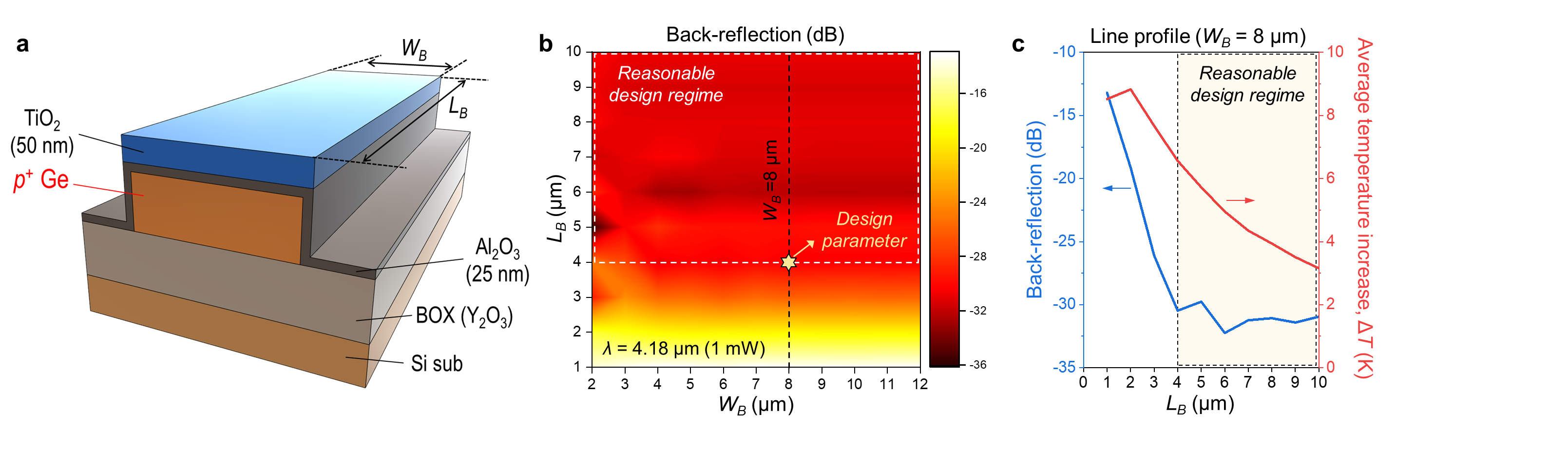


Fig. S4. Optimization of geometrical parameters of the bolometer region. a Schematic of the device structure for simulations. b Simulated contour map showing geometry-dependent back-reflection across varying *L_B_* and *W_B_* of the bolometer region. c Line profiles of both back-reflection and average temperature increase (Δ*T*) within the bolometer region as *L_B_* varies for a fixed *W_B_* of 8 μm.

Figure S4 presents the simulation results used to determine the optimal geometrical parameters of the bolometer region. A schematic of the simulated device structure is illustrated in Fig. S4a. For these simulations, we explored a wide range of lengths (*L_B_*) and widths (*W_B_*) for the bolometer region, while the width of an input waveguide (*W_in_*) leading to the bolometer region was fixed at 2 μm. We first evaluated back-reflection, which can cause unwanted ripples that deteriorate the overall noise characteristics of the sensing system. Figure S4b represents a simulated contour map that shows the geometry-dependent back-reflection. At smaller *L_B_* values, relatively higher back-reflection occurs due to insufficient light absorption in the bolometer region, while changes in *W_B_* have little impact. As shown in Fig. S4b, an *L_B_* greater than 4 μm is preferable, as it ensures back-reflection remains below approximately -30 dB, indicated by a white dotted line and considered within a reasonable design regime. Figure S4c depicts the line profiles of both back-reflection and average temperature increase (Δ*T*) within the bolometer region, as *L_B_* varies for a *W_B_* of 8 μm. Although smaller *W_B_* could potentially enhance heat confinement, a width of 8 μm was chosen due to alignment tolerance constraints in subsequent in-house fabrication steps. Additionally, Δ*T* was calculated within the bolometer region for a central width of 2 μm, corresponding to the spacing of electrodes, due to the minor amount of transfer length between the prepared bolometric material and electrode stack. As shown in Fig. S4c, we finally selected an *L_B_* of 4 μm and a *W_B_* of 8 μm to achieve the highest Δ*T* within the reasonable design regime. It is important to note that the geometrical parameters of the bolometer region can be tailored based on the specific application requirements.

**Supplementary Note 4. Fabrication process flow**


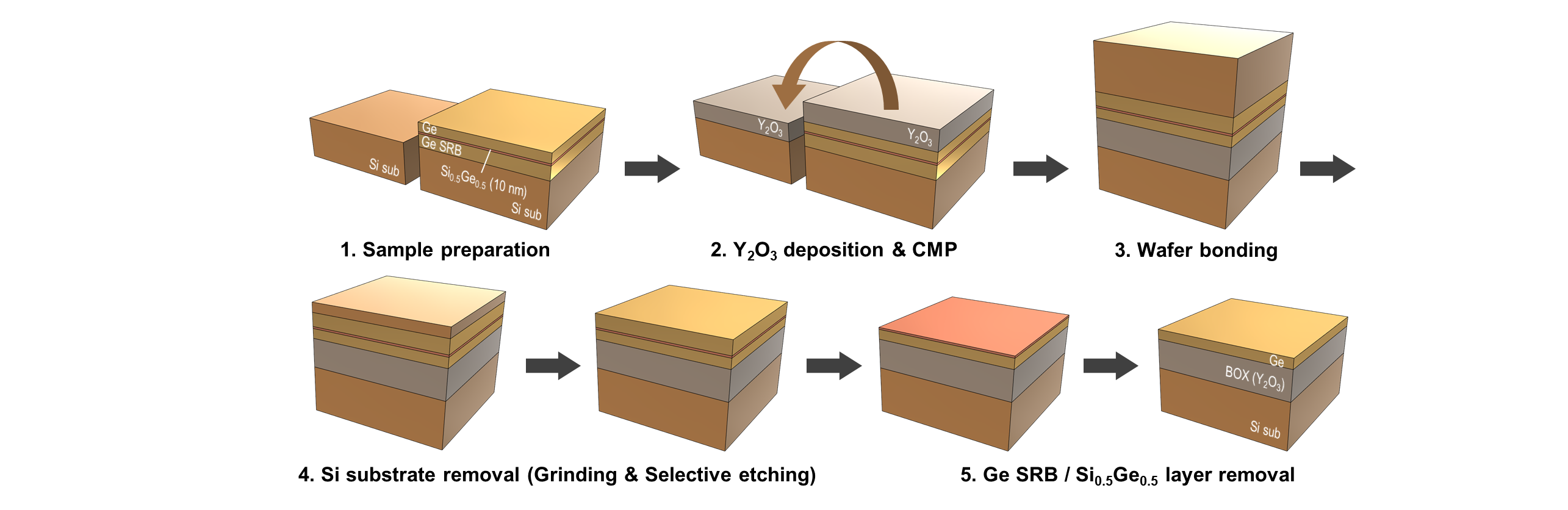


Fig. S5. Fabrication process flow of the Ge-OI photonic platform using direct wafer bonding (DWB) technique. SRB, strain relaxed buffer; BOX, buried oxide; CMP, chemical mechanical polishing.


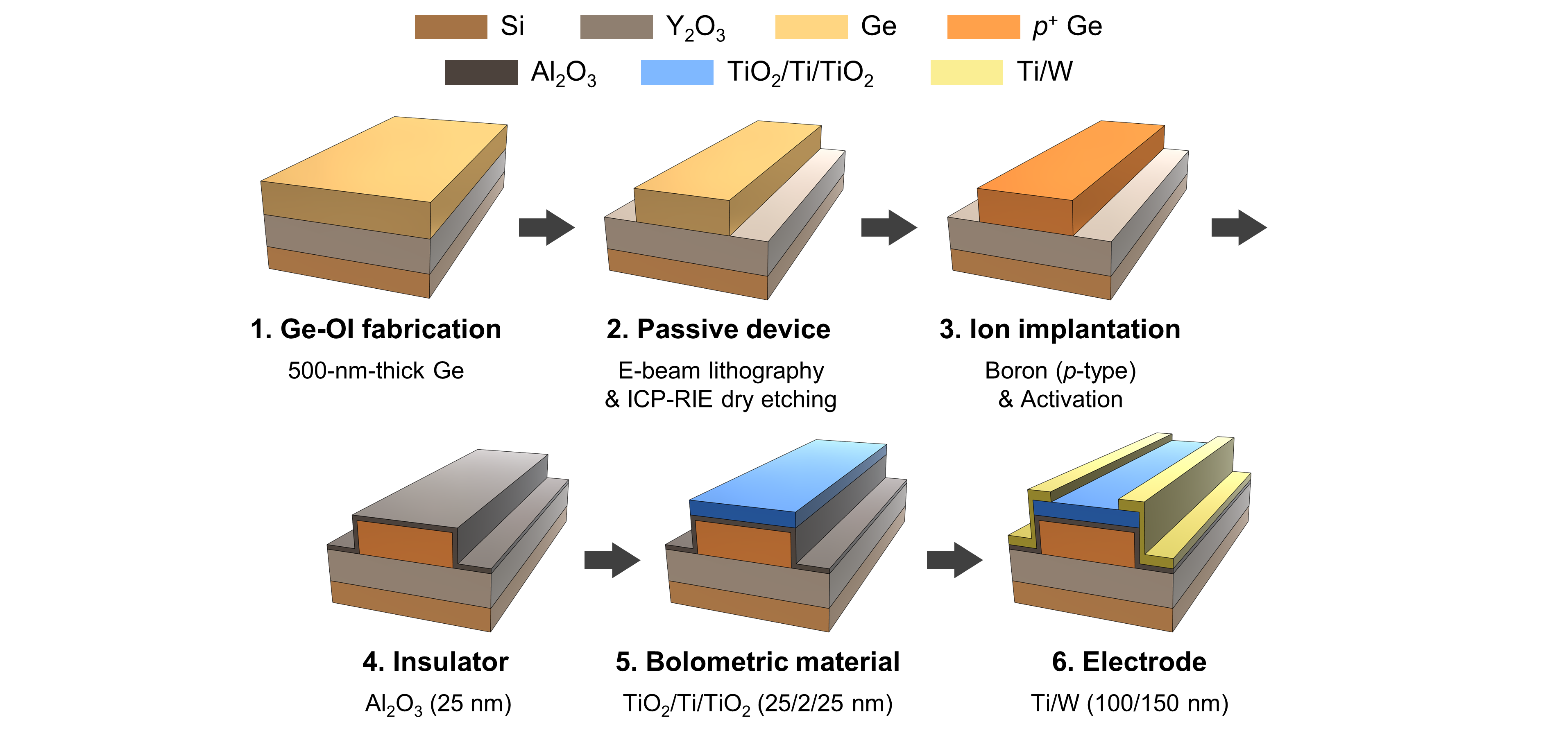


Fig. S6. Fabrication process flow of the proposed MIR waveguide-integrated PD on a Ge-OI platform. ICP-RIE, inductively coupled plasma reactive ion etching.

Figures S5 and S6 summarize the fabrication process flow of the proposed MIR waveguide-integrated photodetector (PD) on the Ge-OI platform using the bolometric effect with FCA process. Our fabrication flow can be seamlessly integrated into standard complementary metal-oxide-semiconductor (CMOS) fabrication workflows, enabling large-scale and high-volume manufacturing with cost-effectiveness. The following conditions were considered: (1) Use of CMOS-compatible materials (Group ІV-based materials) and avoidance of noble metals, which act as contaminants in CMOS fabs, (2) Avoidance of exotic materials that are unavailable or incompatible with standard CMOS fabrication environments, (3) Standard fabrication steps aligned with CMOS processes, including lithography, etching, doping, and deposition techniques, and (4) Processing temperatures that do not exceed the thermal limits of CMOS devices (typically below 500 ºC). For the proof-of-concept demonstration, we adopted electron-beam lithography; however, this could readily be replaced with conventional photolithography since the critical dimension of our device (~200 nm, for slot waveguide structures) is well within the photolithography resolution limits.

**Supplementary Note 5. Device characterization**


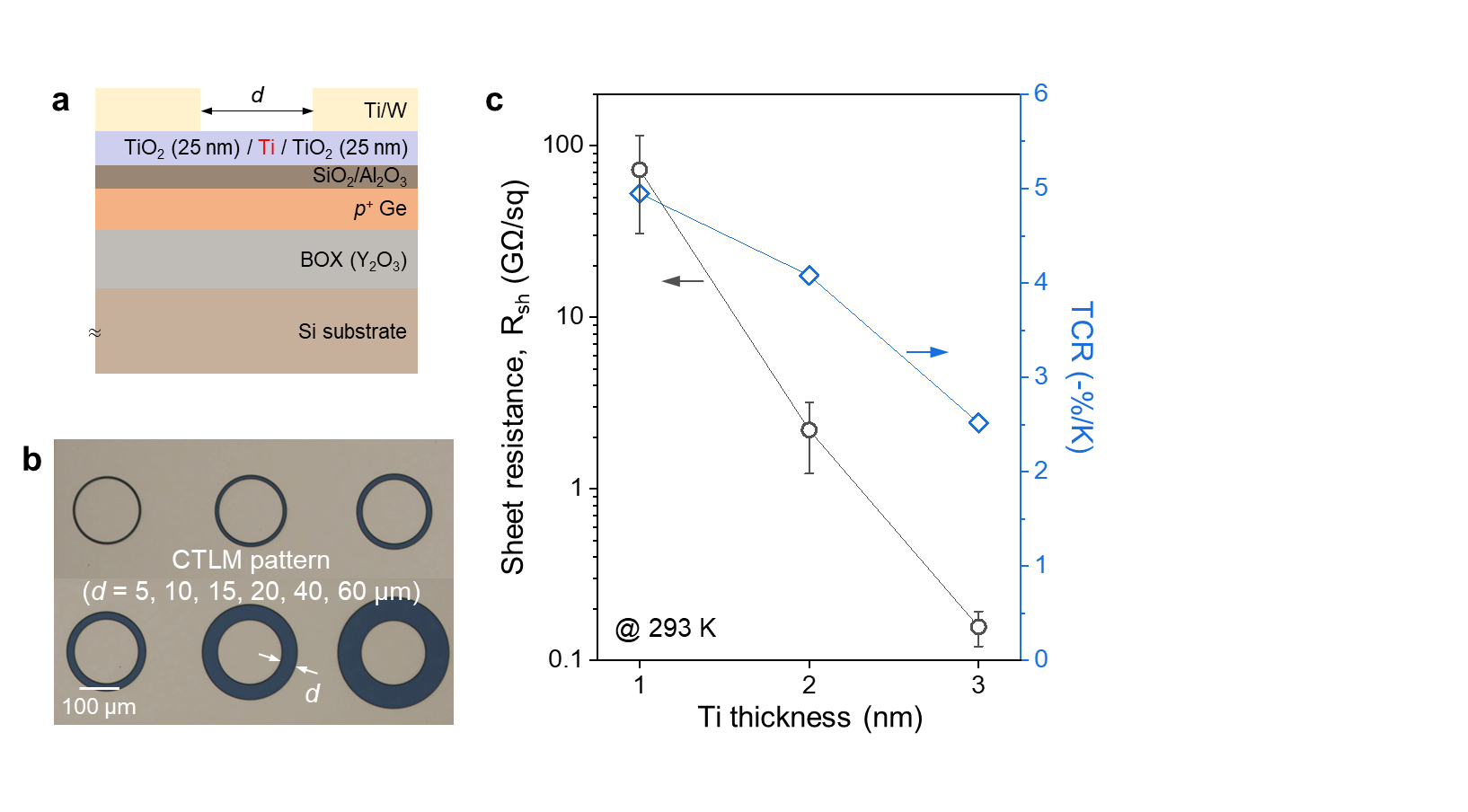


Fig. S7. Optimization of bolometric material. a Schematic of the device structure for the CTLM patterns. b Optical microscope image of the fabricated CTLM patterns. c Variation in sheet resistance (Ω/sq) and TCR (-%/K) depending on the thickness of the metallic Ti layer.

To optimize the thermo-electrical properties of the bolometric material in the TiO_2_/Ti/TiO_2_ tri-layer film, we fabricated the test devices with varying thickness of the metallic Ti layer. Figure S7a illustrates the schematic of these devices, which feature circular transmission line method (CTLM) patterns with spacings (*d*) of 5, 10, 15, 20, 40, and 60 μm, as depicted in the optical microscope image in Fig. S7b. For simplicity, the thickness of the upper and lower TiO_2_ film was maintained at 25 nm, while the thickness of metallic Ti layer varied from 1 to 3 nm. The thickness of the other layers was kept consistent with those used in our waveguide-integrated photodetector. As demonstrated in Fig. S7c, the extracted sheet resistance (R*_sh_*) values from the CTLM patterns at 293 K exhibit a clear downward trend as the thickness of the metallic Ti layer increases. Moreover, the temperature-coefficient of resistance (TCR) values, calculated from the temperature-dependent R*_sh_* values, show a correlation with the R*_sh_* values. Further detailed analysis can be found in our previous work^7^. It is important to note that this approach allows us to tailor the thermo-electrical properties of the bolometric material to meet specific application requirements. Here, we selected a 2 nm thickness for the metallic Ti layer to achieve an optimal balance of electrical resistance and high TCR value in our waveguide-integrated photodetector.


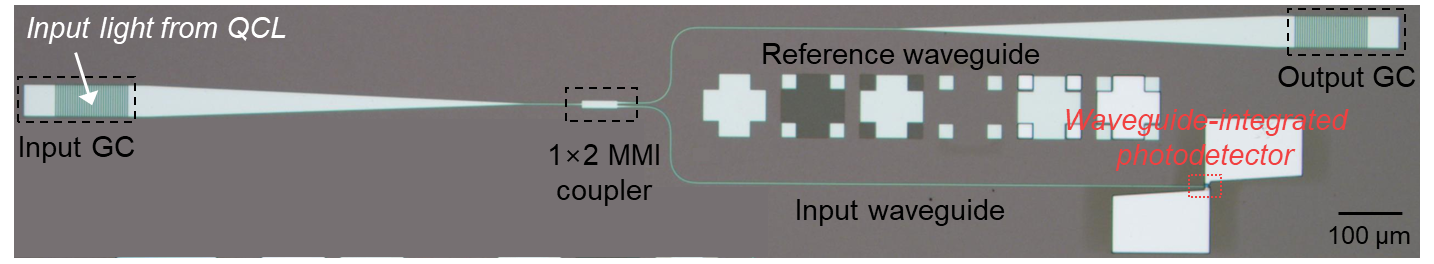


Fig. S8. Optical microscope image of the fabricated device with the reference waveguide pattern. QCL, quantum cascade lasers; GC, grating coupler; MMI, multi-mode interference.

Figure S8 shows an optical microscope image of the fabricated waveguide-integrated photodetector with the reference waveguide structure, which was utilized to assist the optical alignment of fiber-optic coupling and to precisely calculate the optical power coupled into the bolometer region. We characterized the coupling efficiencies of our in-house designed grating couplers (GCs)^8^ by measuring the insertion loss of GC-to-GC structures with a 1-mm-long channel waveguide between GCs. The coupling loss of each GC was determined as half of the insertion loss, after subtracting the propagation loss of 1-mm-long waveguide (Fig. S15a). Subsequently, we measured the insertion loss of the fabricated device from the input GC to the output GC (Fig. S8) and subtracted the coupling loss of one GC facet (output GC) and the propagation loss of the input waveguide. Accounting for this total insertion loss of 10.83 ± 0.14 dB (4.18 µm), the optical power immediately incident on the photodetector (bolometer region) was calibrated.


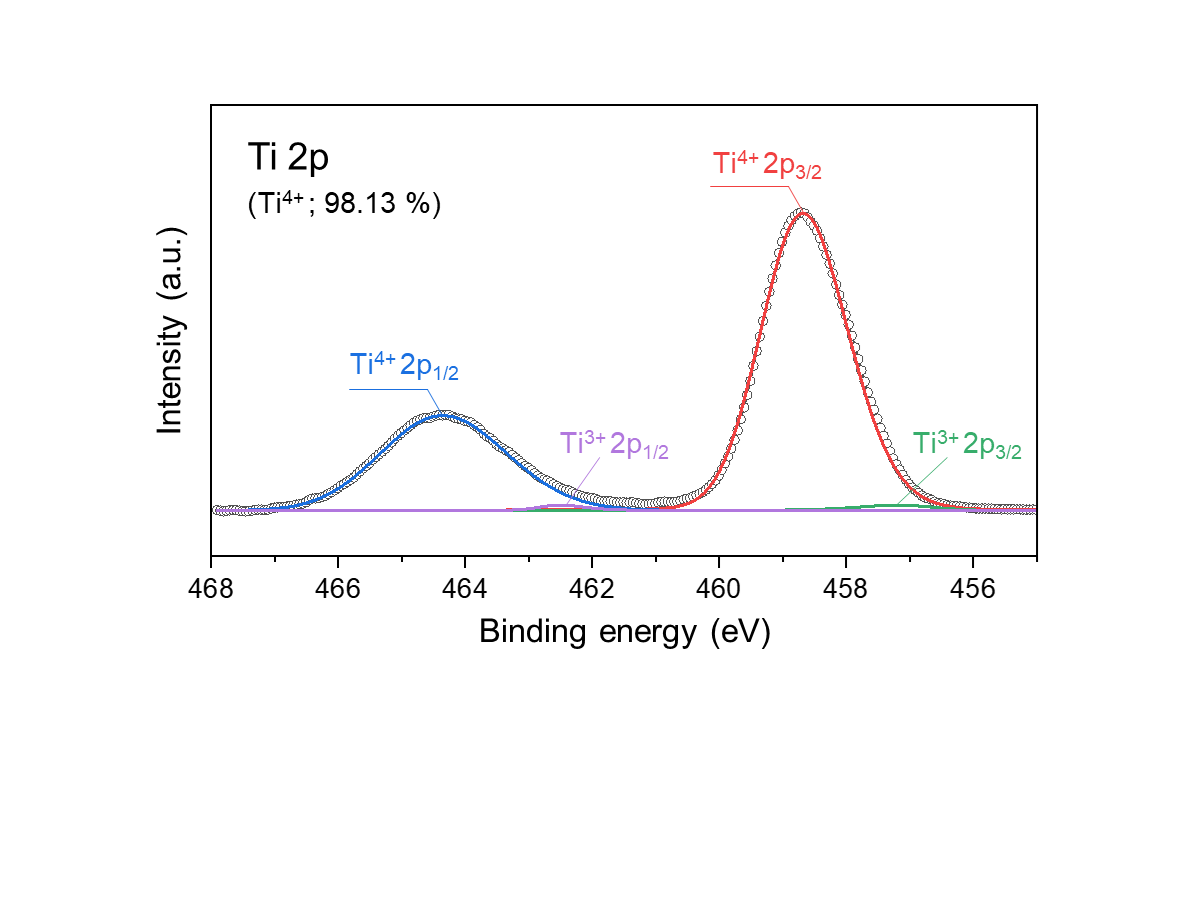


Fig. S9. XPS analysis. The 25 nm-thick TiO_2_ thin film predominantly consists of Ti^4+^ states.

To obtain comprehensive insights into the chemical bonding states and composition of the bolometric material, X-ray photoelectron spectroscopy (XPS) analysis was performed using the Thermo Scientific K-Alpha model at KAIST Analysis Center for Research Advancement (KARA). This analysis employed a monochromatic Al Kα X-ray source to identify the chemical states. Calibration of the energy axis was achieved using the C 1s reference peak. Prior to analysis, the surface of the 25 nm TiO_2_ thin film was cleaned by sputter-cleaning with an Ar^+^ ion beam (~5 nm depth) to prevent the effects of surface contaminants on the XPS results. Figure S9 shows the detailed Ti 2p XPS spectra. Deconvolution of these spectra was performed with reference^9^ to determine the relative proportions of each valence state. The analysis, as shown in Figure S9, indicated that the TiO_2_ thin film predominantly consists of Ti^4+^ states (98.13%), with a minor presence of Ti^3+^ states.


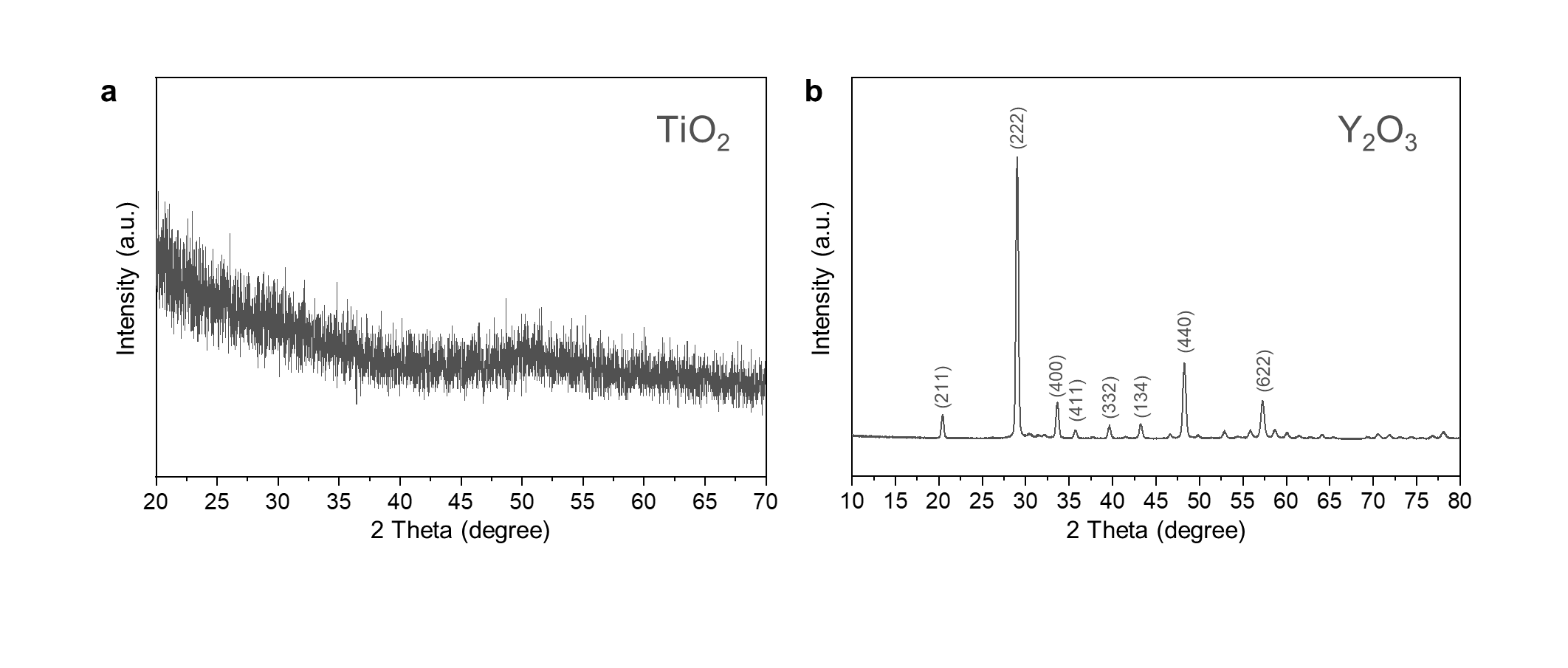


Fig. S10. XRD analysis. a XRD analysis result of the TiO_2_ thin film, showing amorphous state. b XRD analysis result of the Y_2_O_3_ layer, showing polycrystalline state.

To investigate the crystalline properties of both the bolometric material (a 25-nm-thick TiO_2_ film) and the buried oxide (a 2-μm-thick Y_2_O_3_ layer), we performed X-ray diffraction (XRD) analysis using a Cu Kα source in 2-theta scan mode (Rigaku D/MAX-2500 model at KAIST Analysis Center for Research Advancement, KARA). As depicted in Fig. S10a, the diffraction pattern of TiO_2_ film clearly indicated an amorphous state with no sharp peaks, attributed to the insufficient thermal energy required to achieve crystalline phases. In contrast, as shown in Fig. S10b, the sputter-deposited Y_2_O_3_ layer exhibits clear polycrystalline properties, resulting in its relatively higher thermal conductivity of ~7.5 W/(m∙K) (Fig. S2b) compared to typical amorphous state dielectric films^10^.


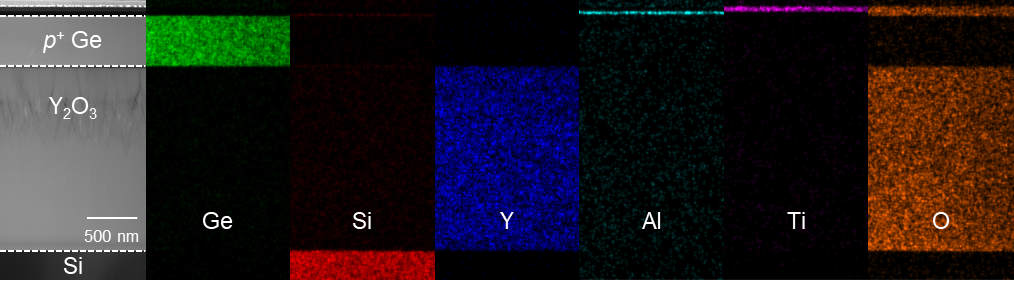


Fig. S11. TEM with EDS analysis. Cross-sectional transmission electron microscopy (TEM) image of the fabricated device with energy-dispersive X-ray spectroscopy (EDS) elemental mapping patterns of Ge, Si, Y, Al, Ti, and O atoms.

**Supplementary Note 6. High-temperature stability**


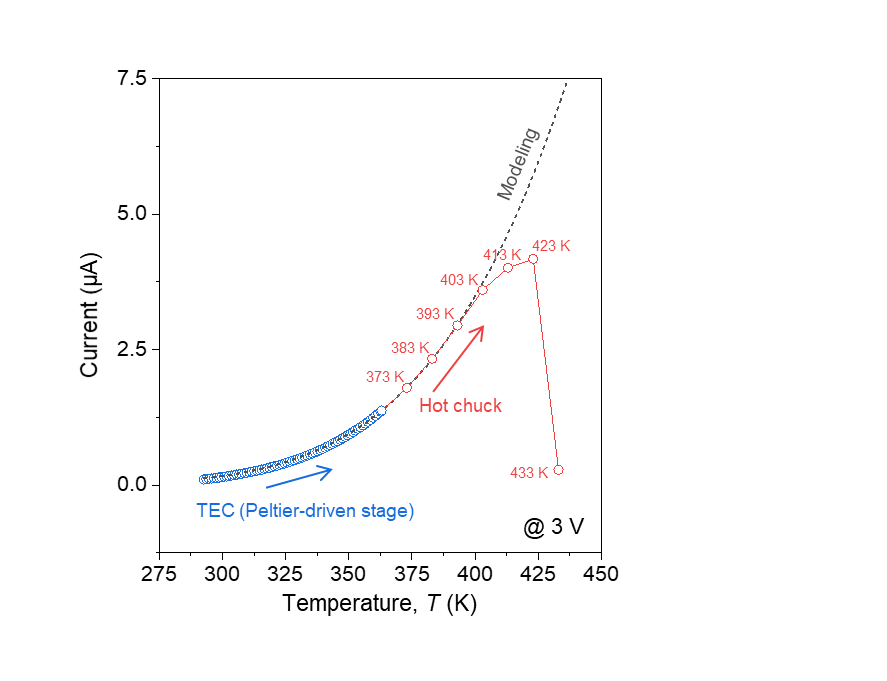


Fig. S12. High-temperature stability. Temperature dependence of current values (3 V) measured from 293 K to 433 K, controlled by a Peltier-driven stage (< 363 K) and a hot chuck (> 363 K). The black dotted line indicates modeled current values depending on the temperature, based on the Arrhenius equation.

Figure S12 presents the temperature-dependent current value at a 3 V bias, measured over the range of 293 K to 433 K. The measurements were conducted using a Peltier-driven stage for the lower temperature regime (< 363 K, as shown in Fig. 2b of the main manuscript) and a hot chuck for the higher temperature regime (> 363 K). As shown in Fig. S12, the measured current values closely follow the modeled current (black dotted line) based on the Arrhenius equation up to ~403 K. However, beyond this threshold temperature, the measured current values begin to diverge from the modeled curve. At 433 K, the current level dropped significantly, and the device could not recover its original resistivity, indicating irreversible changes. This deviation from the modeled curve is attributed to the slight oxidation of the metallic Ti layer within the TiO_2_/Ti/TiO_2_ tri-layer film of the bolometric material^7^. Due to the thin Ti layer (~2 nm), it is susceptible to oxidation, even at relatively lower temperatures, leading to a reduction in conductivity. These oxidation-induced changes in the bolometric material at high temperatures (> 403 K) can affect the maximum optical power that can be used with our detector. Based on the relationship between the thermo-electrical properties (Fig. 2b in the main manuscript) and the photoresponse characteristics (Fig. 3a in the main manuscript), we estimate that the incident optical power corresponding to the threshold temperature (~403 K) is ~97 mW, assuming a linear photoresponse. Further optimization of the thickness of each layer in the bolometric material^7^ and surface passivation strategies with dielectric materials^11^ could enhance high-temperature stability and ultimately elevate the maximum optical power. However, based on our previous work^12^, we believe that the relatively low power regime (a few milliwatts) is adequate for achieving a low limit-of-detection (LoD) in optical gas sensing, and further increases in optical power do not result in significant improvements in the LoD. Thus, the estimated maximum optical power of ~97 mW is sufficient for a wide range of MIR spectroscopy applications.

**Supplementary Note 7. Low-frequency noise analysis**


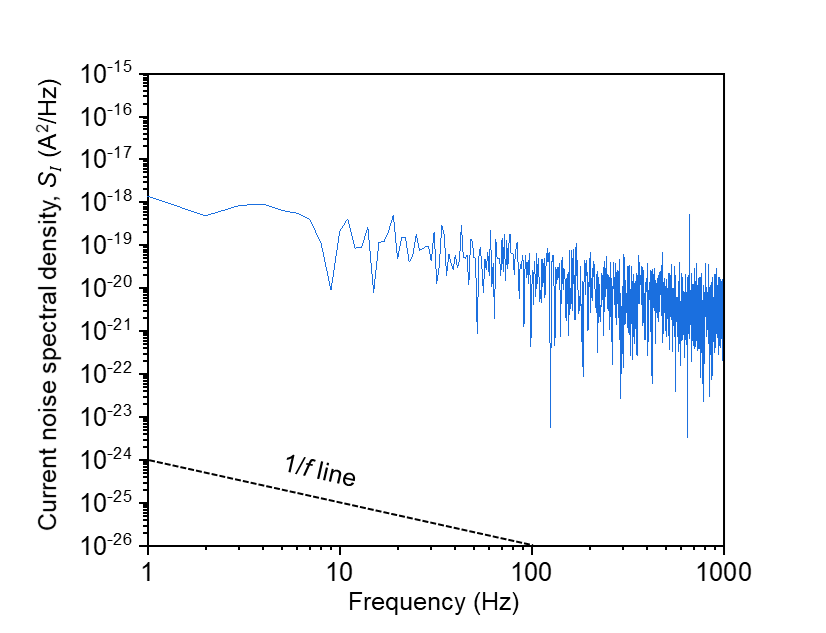


Fig. S13. Low frequency noise (LFN) characteristics. Current noise spectral density, *S_I_* (A^2^/Hz) of the fabricated device as a function of frequency.

Noise characteristics are critical performance metrics for photonic sensing systems. To estimate the noise-equivalent power (NEP) of our device, we examined the low-frequency noise (LFN) features, i.e., current noise spectral density (A^2^/Hz), without light coupling (dark state). As shown in Fig. S13, the noise characteristic predominantly follows the 1/*f* line (dashed line), indicating that the measured noise primarily arises from flicker noise, also known as 1/*f* noise.

**Supplementary Note 8. Electrical breakdown characteristics**

**
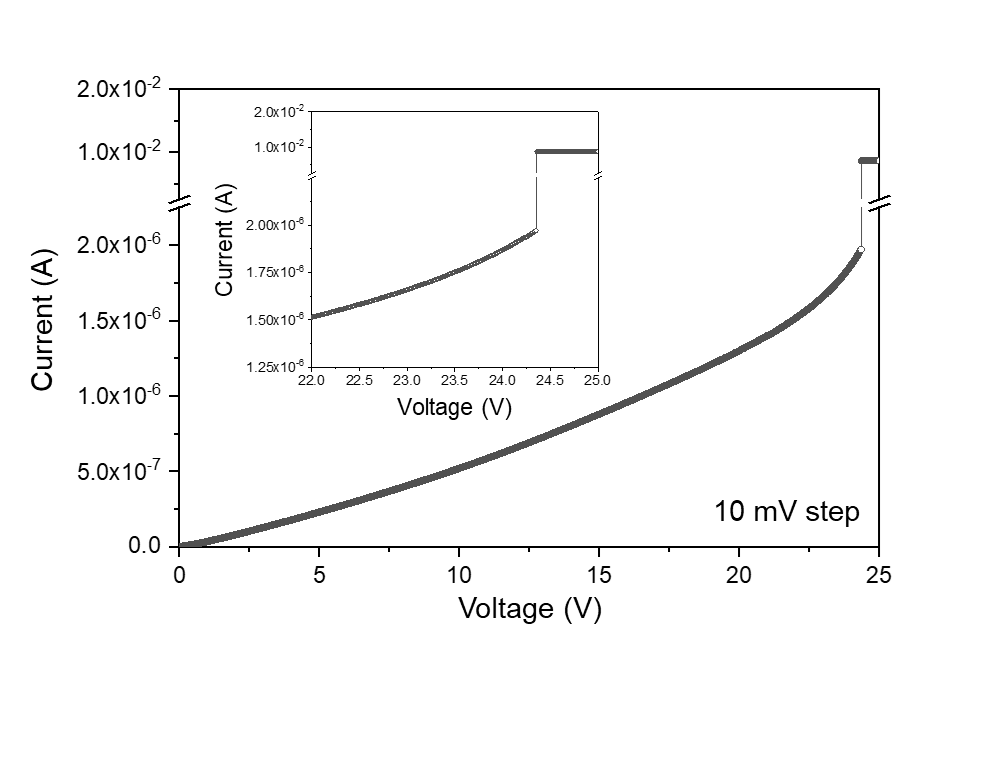
**

Fig. S14. Electrical breakdown. Current-voltage (*I-V*) curve of the fabricated device up to 25 V measured at room temperature with a 10-mV interval, indicating the breakdown voltage of ~23.8 V.

Figure S14 presents the current-voltage (*I-V*) characteristics of our device, which were assessed up to a high voltage range of 25 V at room temperature with a voltage step of 10 mV. It was found that the electrical breakdown occurs at ~23.8 V, characterized by a rapid change in the slope of the *I-V* curve. Beyond this voltage range, the device becomes highly conductive, indicating a transition into the breakdown regime where the device exhibits operational failure.

**Supplementary Note 9. Propagation loss of waveguides**

**
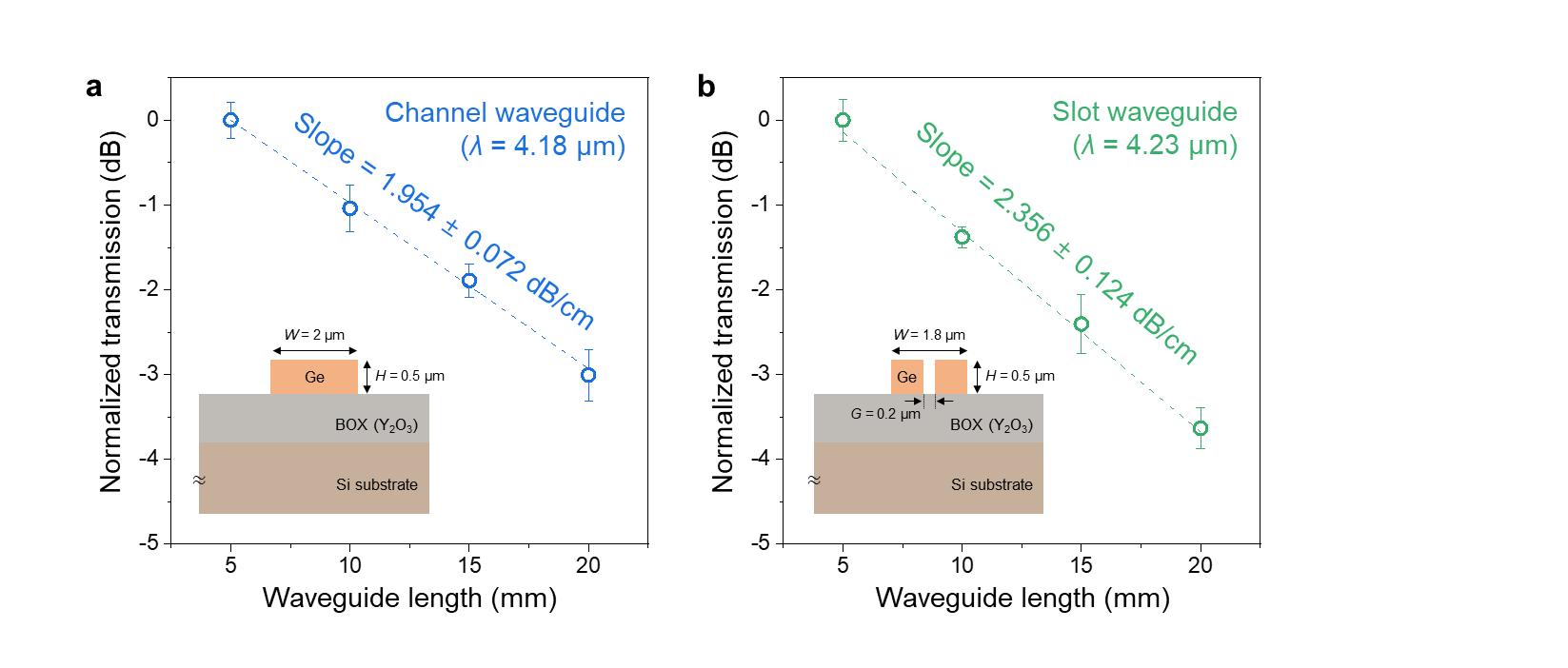
**

Fig. S15. Propagation losses. a Channel waveguide at a wavelength of 4.18 µm. b Slot waveguide at a wavelength of 4.23 µm.

Figure S15a and S15b show the propagation losses for channel and slot waveguides on the Ge-OI platform, respectively, as depicted in each inset. These losses were characterized using the cut-back method under an N_2_ gas purging environment. The results indicate propagation losses of 1.954 ± 0.072 dB/cm for the channel waveguide at a wavelength of 4.18 µm and 2.356 ± 0.124 dB/cm for slot waveguide at 4.23 µm. We found that the propagation losses are relatively large, considering the MIR wavelength range beyond 4 µm. Several strategies can be introduced to reduce the propagation losses: (1) Thermal annealing step with optimized conditions can effectively repair defects in the Ge crystal lattice, such as vacancies and interstitials, caused by electron-beam (e-beam) damage during the e-beam lithography process^13^; (2) Optimization of dry-etching process with SF_6_/C_4_F_8_ chemistry, including gas composition, pressure, and power settings, can achieve smoother sidewalls, thereby mitigating the scattering losses^12^.

**Supplementary Note 10. Simulation of mode converters**

**
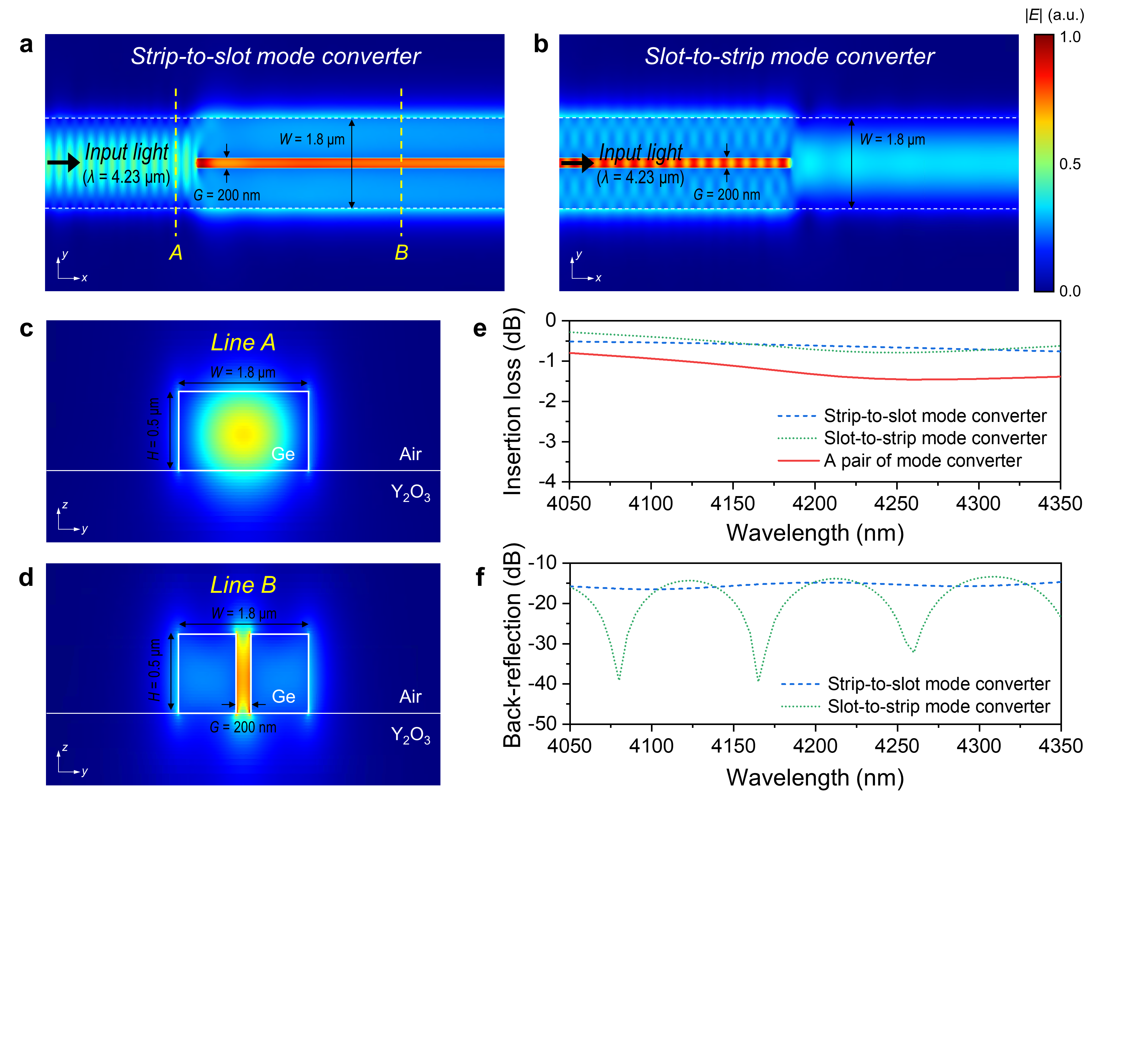
**

Fig. S16. Mode converters on Ge-OI platform. a-b Electric-field distribution for strip-to-slot and slot-to-strip mode converters at a wavelength of 4.23 µm. c-d Cross-sectional field distribution along lines *A* and *B* for the channel and slot waveguide regions, respectively. e Simulated mode conversion efficiencies for individual converters and a pair of mode converter. f Simulated back-reflection of each mode converter.

Figures S16a and S16b depict the electric-field distribution of strip-to-slot and slot-to-strip mode converters on the Ge-OI platform at a wavelength of 4.23 µm in fundamental transverse-electric (TE) mode, respectively. The conversion efficiencies between strip and slot modes^8^ were calculated by a 3D-FDTD solver (Ansys Lumerical). Figure S16c and S16d show the cross-sectional field distribution along lines *A* and *B* in the strip and slot regions, respectively, representing well-confined modal fields. The simulated mode conversion efficiencies for the wavelength range from 4050 to 4350 nm are plotted in Fig. S16e. The insertion of a pair of mode converter at 4.23 µm was estimated to be 1.43 dB. Figure S16f shows the back-reflection of the designed strip-to-slot and slot-to-strip mode converter over the wavelength range from 4050 to 4350 nm, with the values of -14.99 dB and -15.51 dB at 4.23 µm, respectively. Here, multiple back-reflections from the converter facet could result in etalon fringe patterns at the transmission spectrum, which can be further suppressed by carefully designing the mode coupling structure^14,15^.

**Supplementary Note 11. Performance comparison**

Table S1. The overall performance characteristics of the reported MIR waveguide-integrated thermal-type PDs. ChG, chalcogenide glass; BOL, bolometric; PTE, photothermoelectric; TCR, temperature-coefficient of resistance; NEP, noise-equivalent power.

| **Reference, Year** | **Ref** ^16^**, 2019** | **Ref** ^17^**, 2021** | **Ref** ^18^**, 2022** | **Ref** ^19^**, 2024** | **This work** |
| --- | --- | --- | --- | --- | --- |
| **Operation** **wavelength** | 3.72 – 3.88 μm | 3.72 – 3.88 μm | 5.2 μm | 3.61 – 3.7 μm | 4.03 – 4.36 μm |
| **Photonic platform** | Suspended-Si  (c-Si) | Suspended-Si  (a-Si) | ChG-on-CaF_2_  (Ge_28_Sb_12_Se_60_-on-CaF_2_) | Ge-on-Si  (GOS) | Ge-OI  (Ge-on-insulator) |
| **Operation mechanism** | BOL | BOL | PTE | PTE | BOL |
| **Absorption material** | Au | Au | Graphene | Graphene | *p*^+^ Ge |
| **Bolometric material** | a-Si | a-Si | - | - | TiO_2_/Ti/TiO_2_ |
| **TCR (-%/K)** | 0.9 | 1.9 | - | - | 4.262 (@293 K) |
| **Responsivity (%/mW)** | 1.13  (@15 V, 3.8 μm) | 24.62  (@10 V, 3.8 μm) | - | - | 28.77  (@3 V, 4.18 μm) |
| **Responsivity**  **(mA/W)** | 2.26×10^-4^  (@15 V, 3.8 μm) | 2.95×10^-3^  (@10 V, 3.8 μm) | - | - | 3.669×10^-2^  (@3 V, 4.18 μm) |
| **Responsivity, *R***  **(V/W)** | 169.5  (@15 V, 3.8 μm) | 2458.33  (@10 V, 3.8 μm) | 1.5  (@0 V, 5.2 μm) | 1.97  (@0 V, 3.7 μm) | 863.19  (@3 V, 4.18 μm) |
| **Off-state (dark)**  **current (nA)** | 20 (@ 5 V) | 12 (@10 V) | Not stated | Not stated | 127.5 (@3 V) |
| **NEP (W/Hz^0.5^)** | 6.6×10^-5^ | 1.04×10^-5^ | 1.1×10^-9^ | 2.8×10^-9^ | 4.03×10^-7^ |
| ***R* / NEP** | 2.57×10^6^ | 2.36×10^8^ | 1.36×10^9^ | 7.04×10^8^ | 2.14×10^9^ |
| **CMOS-compatibility** | Low | Low | Low | Low | High |

As discussed in this work, a bolometer is a class of thermal-type photodetectors that absorbs incident light, leading to an increase in its temperature. This temperature rise results in a change in the bolometer’s electrical resistance, due to the strong temperature dependence of its resistive material (bolometric material). The change in resistance serves as the primary signal for determining the intensity of incident optical power. There are two main strategies for detecting change in the electrical resistance of the bolometric material: (1) constant voltage source and (2) constant current source, which measure the current and voltage signals, respectively.

In this work, we adopted the constant voltage source method to measure changes in the electrical resistance of the bolometric material. This approach allowed us to measure the current variation corresponding to varying incident optical power. Here, we introduced the responsivity in terms of ‘%/mW’ to represent the performance of bolometric photodetection, which indicates the percentage change in current as a function of incident optical power. This unit provides a fairer basis for comparison, particularly for bolometric detectors. The conventional unit (A/W or V/W) can vary significantly depending on the electrical resistivity of the bolometric material (resistive film) and the magnitude of operating source (voltage or current), which may lead to misunderstandings of the detector’s performance. However, for a comprehensive comparison with previous literatures, as shown in Table S1, we utilized the relationship between responsivity values expressed in different units. Details of these calculations are described below.

*Derivation Steps*

The definition of variables used for this derivation steps are below:

*S* [%/mW]: Percentage responsivity in current (percentage change of current as a function of optical power)

*I_off_* [A]: Off-state current (dark current)

*R_I_* [A/W]: Current responsivity

*R_V_* [V/W]: Voltage responsivity

*P_in_* [W]: Incident optical power

*R* [Ω]: Electrical resistance

*V* [V]: Voltage

*I* [A]: Current

We aim to derive the relationship between the voltage responsivity (*R_V_*) and current responsivity (*R_I_*) expressed as in Eq. (1):

$$R_{V}\text{ }[\text{V/W}]=-R\text{ }[\Omega]\text{ }\times\text{ }R_{I}\text{ }[\text{A/W}]$$

The voltage change (Δ*V*) under constant current bias (*I_bias_*) can be described as:

$$\Delta V=I_{bias}\times\Delta R$$

And, the current change (Δ*I*) under the constant voltage bias (*V_bias_*) is:

$$\Delta I=-\frac{V_{bias}}{R^{2}}\times\Delta R$$

(Detailed derivation of Eq. (3) is provided separately below.)

From the definition of current responsivity (*R_I_*) and Eq. (3):

$$R_{I}=\frac{\Delta I}{P_{in}}=-\frac{V_{bias}}{R^{2}}\times\frac{\Delta R}{P_{in}}$$

From Eq. (4):

$$\frac{\Delta R}{P_{in}}=-\frac{R^{2}}{V_{bias}}\times R_{I}$$

Substituting back into the definition for *R_V_* and from Eqs. (2) and (5):

$$R_{V}=\frac{\Delta V}{P_{in}}=I_{bias}\times\frac{\Delta R}{P_{in}}=I_{bias}\times\left(-\frac{R^{2}}{V_{bias}}\times R_{I}\right)$$

Simplify Eq. (6):

$$R_{V}=-\frac{I_{bias}R^{2}}{V_{bias}}\times R_{I}$$

With the relationship for *I_bias_*:

$$I_{bias}=\frac{V_{bias}}{R}$$

From Eqs. (7) and (8), we finally have:

$$R_{V}=-R\times R_{I}$$

In addition, from the definition for current responsivity (*R_I_*), the relationship between *R_I_* and *S* is expressed as:

$$R_{I}=\frac{\Delta I}{P_{in}}=I_{off}\left(1+\frac{S}{100}\times1000\right)-I_{off}=I_{off}\times10\times S$$

Consequently, we can obtain the relationship of the Eq. (11) from Eqs. (9) and (10):

$$R_{V}\text{ } [\text{V/W}]=-R\text{ }[\Omega]\text{ }\times\text{ }I_{off}\text{ }[\text{A}]\text{ }\times\text{ }10\text{ }\times\text{ }S\text{ }[\text{\%/mW}]$$

*Additional Derivation Steps*

Here, we aim to derive the Eq. (12):

$$\Delta I=-\frac{V_{bias}}{R^{2}}\times\Delta R$$

Under constant voltage bias, the current through our bolometric photodetector is given by *Ohm’s Law*:

$$I=\frac{V_{bias}}{R}$$

After the resistance change (Δ*R*) due to light injection, the current becomes:

$$I+\Delta I=\frac{V_{bias}}{R+\Delta R}$$

$$\Delta I=\left(\frac{V_{bias}}{R+\Delta R}\right)-\left(\frac{V_{bias}}{R}\right)$$

Simplify this expression:

$$\Delta I=V_{bias}\left(\frac{1}{R+\Delta R}-\frac{1}{R}\right)$$

Here, we can consider *I* as a function of *R*:

$$I(R)=\frac{V_{bias}}{R}$$

Differentiating *I* with respect to *R*:

$$\frac{dI}{dR}=-\frac{V_{bias}}{R^{2}}$$

For small changes, Δ*I* can be expressed as:

$$\Delta I\approx\frac{dI}{dR}\Delta R$$

Thus, from Eqs. (18) and (19) we can finally get the Eq. (20), which aligns with Eq (3):

$$\Delta I=-\frac{V_{bias}}{R^{2}}\times\Delta R$$

**References**

1. Zhang, L. et al. The Franz-Keldysh effect and free carrier dispersion effect in germanium. *Optik* **159**, 202-210 (2018).

2. Nedeljkovic, M., Soref, R. & Mashanovich, G. Z. Predictions of free-carrier electroabsorption and electrorefraction in germanium. *IEEE Photonics Journal* **7**, 2600214 (2015).

3. Shen, L. et al. Two-photon absorption and all-optical modulation in germanium-on-silicon waveguides for the mid-infrared. *Optics Letters* **40**, 2213 (2015).

4. Olson, D. H., Braun, J. L. & Hopkins, P. E. Spatially resolved thermoreflectance techniques for thermal conductivity measurements from the nanoscale to the mesoscale. *Journal of Applied Physics* **126**, 150901 (2019).

5. Kakefuda, Y. et al. Thermal conductivity of PrRh_4.8_B_2_, a layered boride compound. *APL Materials* **5**, 126103 (2017).

6. Baba, T. Analysis of one-dimensional heat diffusion after light pulse heating by the response function method. *Japanese Journal of Applied Physics* **48**, 05EB04 (2009).

7. Shim, J. et al. Tailoring bolometric properties of a TiO_x_/Ti/TiO_x_ tri-layer film for integrated optical gas sensors. *Optics Express* **29**, 18037 (2021).

8. Lim, J. et al. Low-loss and high-confinement photonic platform based on germanium-on-insulator at mid-infrared range for optical sensing. *Journal of Lightwave Technology* **41**, 2824-2833 (2023).

9. Biesinger, M. C., Lau, L. W. M., Gerson, A. R. & Smart, R. St. C. Resolving surface chemical states in XPS analysis of first row transition metals, oxides and hydroxides: Sc, Ti, V, Cu and Zn. *Applied Surface Science* **257**, 887-898 (2010).

10. Scott, E. A., Gaskins, J. T., King, S. W. & Hopkins, P. E. Thermal conductivity and thermal boundary resistance of atomic layer deposited high- *k* dielectric aluminum oxide, hafnium oxide, and titanium oxide thin films on silicon. *APL Materials* **6**, 058302 (2018).

11. Gupta, B. *et al.* Recent advances in materials design using atomic layer deposition for energy applications. *Advanced Functional Materials* **32**, 2109105 (2022).

12. Lim, J. et al. Ultrasensitive mid-infrared optical gas sensor based on germanium-on-insulator photonic circuits with limit-of-detection at sub-ppm level. *ACS Photonics* **11**, 4268-4278 (2024).

13. Shams-Ansari, A. et al. Reduced material loss in thin-film lithium niobate waveguides. *APL Photonics* **7**, 081301 (2022).

14. Yallew, H. D. *et al.* Sub-ppm methane detection with mid-infrared slot waveguides. *ACS Photonics* **10**, 4282-4289 (2023).

15. Deng, Q., Liu, L., Li, X. & Zhou, Z. Strip-slot waveguide mode converter based on symmetric multimode interference. *Optics Letters* **39**, 5665 (2014).

16. Wu, Y. et al. Mid-infrared nanometallic antenna assisted silicon waveguide based bolometers. *ACS Photonics* **6**, 3253-3260 (2019).

17. Wu, Y. et al. Nanometallic antenna-assisted amorphous silicon waveguide integrated bolometer for mid-infrared. *Optics Letters* **46**, 677 (2021).

18. Goldstein, J. et al. Waveguide-integrated mid-infrared photodetection using graphene on a scalable chalcogenide glass platform. *Nature Communications* **13**, 3915 (2022).

19. Cai, H. et al. Mid-infrared waveguide-integrated and photo-thermoelectric graphene photodetector based on germanium-on-silicon platform. *APL Photonics* **9**, 096101 (2024).
